# Modulating the Microbiota as a Therapeutic Intervention for Type 2 Diabetes

**DOI:** 10.3389/fendo.2021.632335

**Published:** 2021-04-07

**Authors:** M. Nazmul Huda, Myungsuk Kim, Brian J. Bennett

**Affiliations:** ^1^ Department of Nutrition, University of California Davis, Davis, CA, United States; ^2^ Obesity and Metabolism Research Unit, United States Department of Agriculture (USDA), Agricultural Research Service (ARS), Western Human Nutrition Research Center, Davis, CA, United States

**Keywords:** microbiota (16S), type 2 diabetes (T2D), metabolites, probiotics, prebioitcs, intermittent fasting, genetics, epigenetics

## Abstract

Mounting evidence suggested that the gut microbiota has a significant role in the metabolism and disease status of the host. In particular, Type 2 Diabetes (T2D), which has a complex etiology that includes obesity and chronic low-grade inflammation, is modulated by the gut microbiota and microbial metabolites. Current literature supports that unbalanced gut microbial composition (dysbiosis) is a risk factor for T2D. In this review, we critically summarize the recent findings regarding the role of gut microbiota in T2D. Beyond these associative studies, we focus on the causal relationship between microbiota and T2D established using fecal microbiota transplantation (FMT) or probiotic supplementation, and the potential underlying mechanisms such as byproducts of microbial metabolism. These microbial metabolites are small molecules that establish communication between microbiota and host cells. We critically summarize the associations between T2D and microbial metabolites such as short-chain fatty acids (SCFAs) and trimethylamine N-Oxide (TMAO). Additionally, we comment on how host genetic architecture and the epigenome influence the microbial composition and thus how the gut microbiota may explain part of the missing heritability of T2D found by GWAS analysis. We also discuss future directions in this field and how approaches such as FMT, prebiotics, and probiotics supplementation are being considered as potential therapeutics for T2D.

## Introduction

Diabetes is a metabolic disorder characterized by elevated blood glucose levels. The incidence of diabetes is widespread and the International Diabetes Federation (IDF) reports that 463 million people in the world are suffering from diabetes, which is estimated to reach 700 million by the year 2045 (1). In the USA, 13% of adults aged 18 or older have diabetes (2). Diabetes has been categorized into three classes (type 1, type 2, and gestational diabetes) depending on the underlying pathophysiology. Among them, type 2 diabetes (T2D) accounts for more than 90% of all diabetes (1, 2). Though genetic susceptibility is a critical determinant of T2D, non-genetic factors, including diet, physical activity, also play a significant role in the development and severity of T2D. The recent understanding that microbiota is a critical determinant of human health has opened a new avenue of basic and clinical research for T2D.

The microbiota refers to an assemblage of living microorganisms including bacteria, archaea, and fungi present in a defined environment (3). The microbiota can reside within or on the host and can modulate nutritional status, health, and diseases of the host (4). The most widely used technique to assay microbiota is to quantify the variable region(s) of the 16S rRNA gene (5). 16S rRNA gene analysis is economical and straightforward, however, it provides a limited regulation of taxonomic information, up to the genus level reliably. Alternatively, metagenomics, a whole-genome shotgun sequencing approach of all the DNA present in the sample, provides much better taxonomic resolution down to species or strain level. Additionally, metagenomics encompasses the collective functional genomes of all microorganisms, thus provides an opportunity for functional profiling of the metabolic pathways present in a community (6). The diversity and composition of the gut bacteria have been intensely studied, as well as their impact on health and diseases (7), including obesity (8), inflammation (9), and T2D (10). A better understanding of the link between the gut microbiota and metabolic disorders, especially T2D, may lead to advances in current treatment approaches, accurate disease monitoring, and development of novel therapeutics.

In addition to sex and age, both diet and the immune system contribute to the composition of the microbiota (11). The underlying architecture of the host’s genetics may also shape the community structure of the gut microbiota (12). Several genetic variants are associated with T2D susceptibility (13) and it is speculated that a part of “missing heritability” described in genome-wide association study (GWAS) studies (14) may be explained by gut microbiota. Moreover, growing evidence suggests that gut microbial metabolites regulate gene expression through a variety of classic signaling pathways (14) and more recently epigenetics (15). Thus, understanding the complex interactions between microbiome, microbial metabolome, and host genome will assist the development of novel therapeutics.

In this review, we critically summarize the recent developments describing the role of microbiota on T2D susceptibility, development, and severity. In particular, we focus on the underlying biochemical mechanisms by which gut microbiota may affect T2D. A number of these mechanisms may be mediated by the host genetics, and epigenetics thus may be viable targets for precision medicine. The potential effects of prebiotics, probiotics, medication, and intermittent fasting on the microbiota and T2D are extensively discussed. Finally, we comment on the future direction in this field.

## Dysbiosis, Obesity, Low-Grade Inflammation and T2D

**Figure 1:**
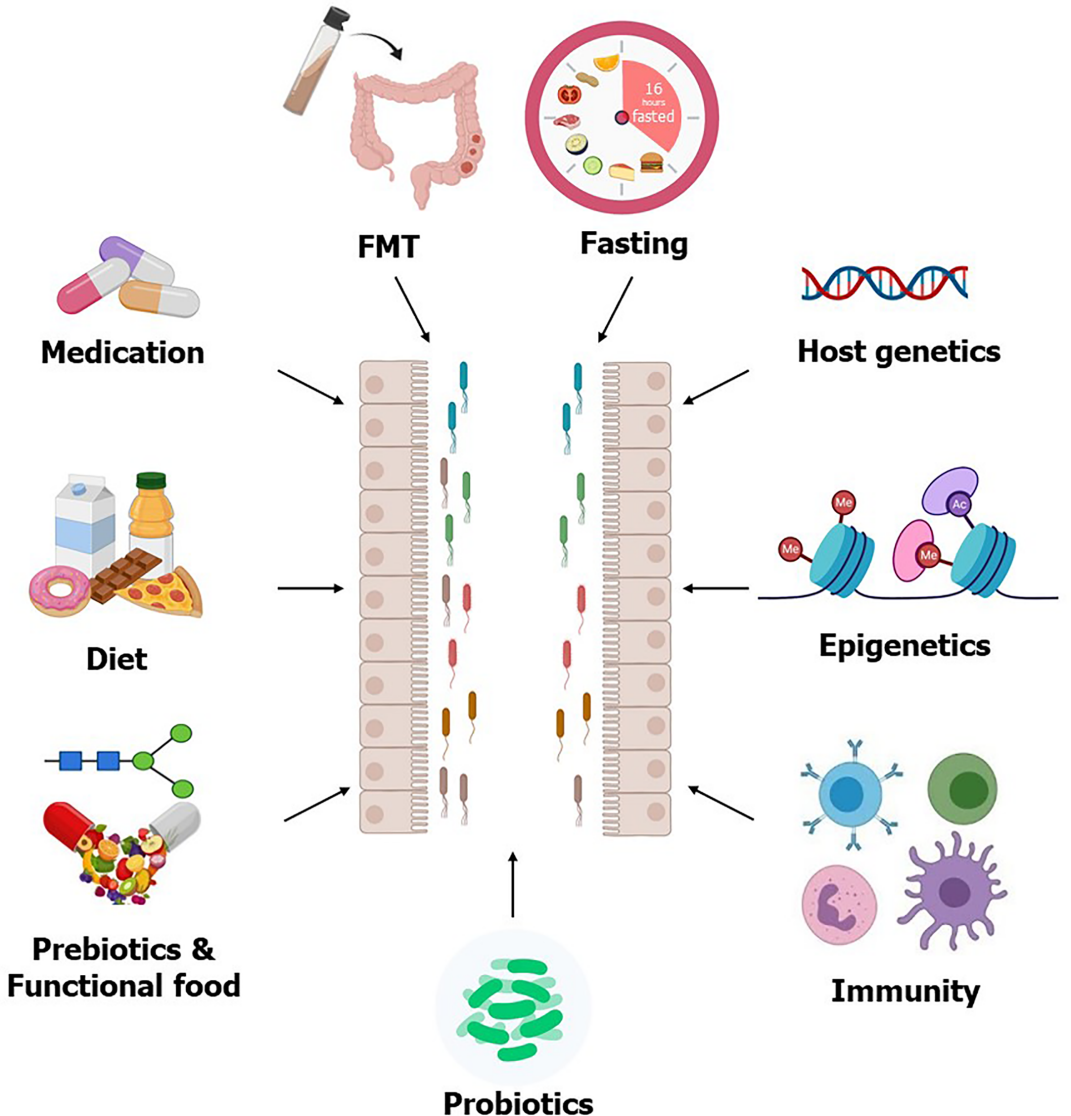
**Factors affecting gut microbiota**. The gut microbial composition can be modulated by different interventions such as prebiotics, probiotics, FMT, and intermittent fasting, all of which are considering as potential therapeutics for T2D. Host genetics, epigenetics, and immunity also modulate gut microbiota. Some T2D medication improves circulating glucose levels partly through modulating gut microbiota, which further supports the usability of the gut microbiota as therapeutics for T2D.

A growing number of studies suggest that gut microbiota influences T2D susceptibility, development, severity, and progression. Dysbiosis, an alteration of a healthy microbiota, is associated with obesity, low-grade inflammation, insulin resistance, and T2D which potentially reflects a causal role linking these pathologies (16). Along with animal studies, numerous human cohorts also have reported specific gut bacteria enriched or depleted in T2D patients compared to healthy controls. A summary of the recent reports of altered microbiota found in T2D patients is depicted in **Table 1**, and the interactions between environmental factors, genetics, microbiota, microbial metabolites, obesity, inflammation, and T2D are shown in **Figure 1**.

**Table 1:** T2D-related gut microbiota found in human studies.

Sample size	Age	Sex	Technique	Associated microbiota changes	References
183 T2D 185 Controls (Chinese)	13–86	Women (153) Men (209)	Metagenomic sequencing	Increased in T2D: *Akkermansia muciniphila*, *Bacteroides caccae, Clostridium hathewayi, Clostridium ramosum, Clostridium symbiosum, Desulfovibrio* sp.*, Eggerthella lenta*, and *Escherichia coli*	Qin et al. (17)
				Decreased in T2D: *Clostridiales* sp. *SS3/4, Eubacterium rectale, Faecalibacterium prausnitzii, Roseburia intestinalis*, and *Roseburia inulinivorans* **Significantly correlated bacteria with T2D related traits: *Roseburia intestinalis* (-), *Faecalibacterium prausnitzii* (-), *Akkermansia muciniphila* (-), *Desulfovibrio* (-), *Bacteroides caccae* (+).**	
53 T2D 49 Impaired glucose tolerance 43 Controls (Swedish females)	69–72	Women (145)	Metagenomic sequencing	Increased in T2D: *Clostridium clostridioforme, Lactobacillus gasseri*, and *Streptococcus mutans*	Karlsson et al. (18)
				Decreased in T2D: *Roseburia, Clostridium* spp.*, Eubacterium eligens*, and *Bacteroides intestinalis* **Significantly correlated bacteria with T2D related traits: *Roseburia intestinalis* (-), *Faecalibacterium prausnitzii* (-), *Akkermansia muciniphila* (-), *Bacteroides intestinalis* (-), *Clostridium clostridioforme* (+)*, Lactobacillus gasseri* (+).**	
75 T2D, 291 Controls (Danish)	50–66	Women (187) Men (179)	Metagenomic sequencing	Increased in T2D: BCAA-producing bacteria *(Prevotella copri and Bacteroides vulgatus)*	Pedersen et al. (19)
				Decreased in T2D: *Faecalibacterium, Oscillibacter, Roseburia, Bifidobacterium, Coprococcus*, and *Butyrivibrio* **Significantly correlated bacteria with T2D related traits: *Faecalibacterium prausnitzii* (-)*, Akkermansia muciniphila* (-)*, Bacteroides vulgatus* (+)*, Prevotella copri* (+), and *Clostridia* sp.**	
46 T2D, 75 Combined glucose intolerance 178 Impaired glucose tolerance 189 Impaired fasting glucose 523 Controls (Swedish)	57–61	Women (568) Men (443)	Metagenomic sequencing	Increased in T2D: *Coprococcus eutactus, Clostridiales bacterium*, and *Lachnospiraceae bacterium*	Wu et al. (20)
				Decreased in T2D: *Clostridium* sp.*, Clostridium hathewayi, Clostridium bolteae, Clostridium symbiosum*, and *Roseburia faecis*	
13 T2D, 64 Prediabetes 44 Controls (Chinese)	52–55	NA	16S rRNA V3-V5 region	Increased in T2D: *Clostridia, Collinsella, Dorea, Prevotella, Ruminococcus*, and *Verrucomicrobia*	Zhang et al. (21)
				Decreased in T2D: *Bacteroides, Akkermansia muciniphila, Faecalibacterium prausnitzii, Roseburia*, and *Streptococcus*	
20 T2D, 40 Controls (Chinese)	NA	Women (42) Men (18)	16S rRNA V4-V5 region	Increased in T2D: *Streptococcus, Dorea*, and *Fusobacterium*	Li et al. (22)
				Decreased in T2D: *Akkermansia, Bifidobacterium, Faecalibacterium*, and *Parabacteroides*	
98 T2D, 193 Controls (Nigerian)	41–70	NA	16S rRNA V4 region	Increased in T2D: *Bacteroidetes, Prevotella, Desulfovibrio piger, Eubacteriu*, and *Peptostreptococcus*	Doumatey et al. (23)
				Decreased in T2D: *Anaerostipes, Ruminococcus, Cellulosilyticum ruminicola, Clostridium paraputrificum, Clostridium butyricum, Collinsella*, and *Epulopiscium*	
18 T2D, 18 Controls (Danish males)	31–73	Men (36)	16S rRNA V4 region	Increased in T2D: Betaproteobacteria	Larsen et al. (24)
				Decreased in T2D: Firmicutes and Clostridia	
134 T2D, 37 Controls (Chinese)	45–67	Women (92) Men (79)	16S rRNA V3-V4 region	Increased in T2D: *Prevotella, Dialister*, and *Sutterella*	Wang et al. (25)
				Decreased in T2D: *Bacteroides, Bifidobacterium, Clostridium XIVa, Parabacteroides, Staphylococcus, Granulicatella, Porphyromonas, Clostridium XI, Blautia, Anaerostipes, Clostridium XVIII, Fusicatenibacter, Enterococcus, Clostridium IV, Eggerthella*, and *Flavonifractor.*	
22 T1D, 23 T2D, 23 Controls (Polish)	20–65	Women (40) Men (28)	16S rRNA	Increased: Firmicutes/Bacteroidetes ratio, Verrucomicrobia, Ruminococcus	Salamon et al. (26)
				Decreased: *Bacteroides*, *Roseburia* and *Faecalibacterium*	

Landmark studies in the 2000s (27–29) demonstrated that the microbiota contributes to digestion, carbohydrate metabolism, obesity, and plasma glucose levels. Additionally, those studies established a causal relationship by showing that the susceptibility to obesity could be transferred between mice when the fecal microbiota of obese mice was transplanted into non-obese animals. Consistent with these findings, several other studies have reported enrichment or depletion of specific obesity-related gut bacteria and indicated a connection between gut microbiota, adiposity, and T2D. For example, increased abundance of *Prevotella* and decreased abundance of *Bacteroides* were associated with a higher risk of obesity with metabolic syndrome, while body mass index and body fat percentage were negatively correlated with *Coprococcus* abundance (30). More recent data suggests that the abundance of a bacteria in the Bacilli family was positively associated with fat mass, and negatively associated with lean mass and plasma glucose level (31). Additionally, *Peptostreptococcaceae*, *Blautia*, and a bacterium related to the *Clostridiaceae* family were positively associated with plasma glucose levels (31). In a recent randomized, double-blind, placebo-controlled clinical trial with overweight or obese insulin-resistant subjects, pasteurized *Akkermansia muciniphila* supplementation was associated with weight loss, improved insulin sensitivity, and reduced insulinemia (32). A potential mechanism of these positive effects is an interaction between temperature stable outer membrane protein Amuc 1100 found in pasteurized *Akkermansia muciniphila* and Toll-like receptor 2 (33).

**Figure 2:**
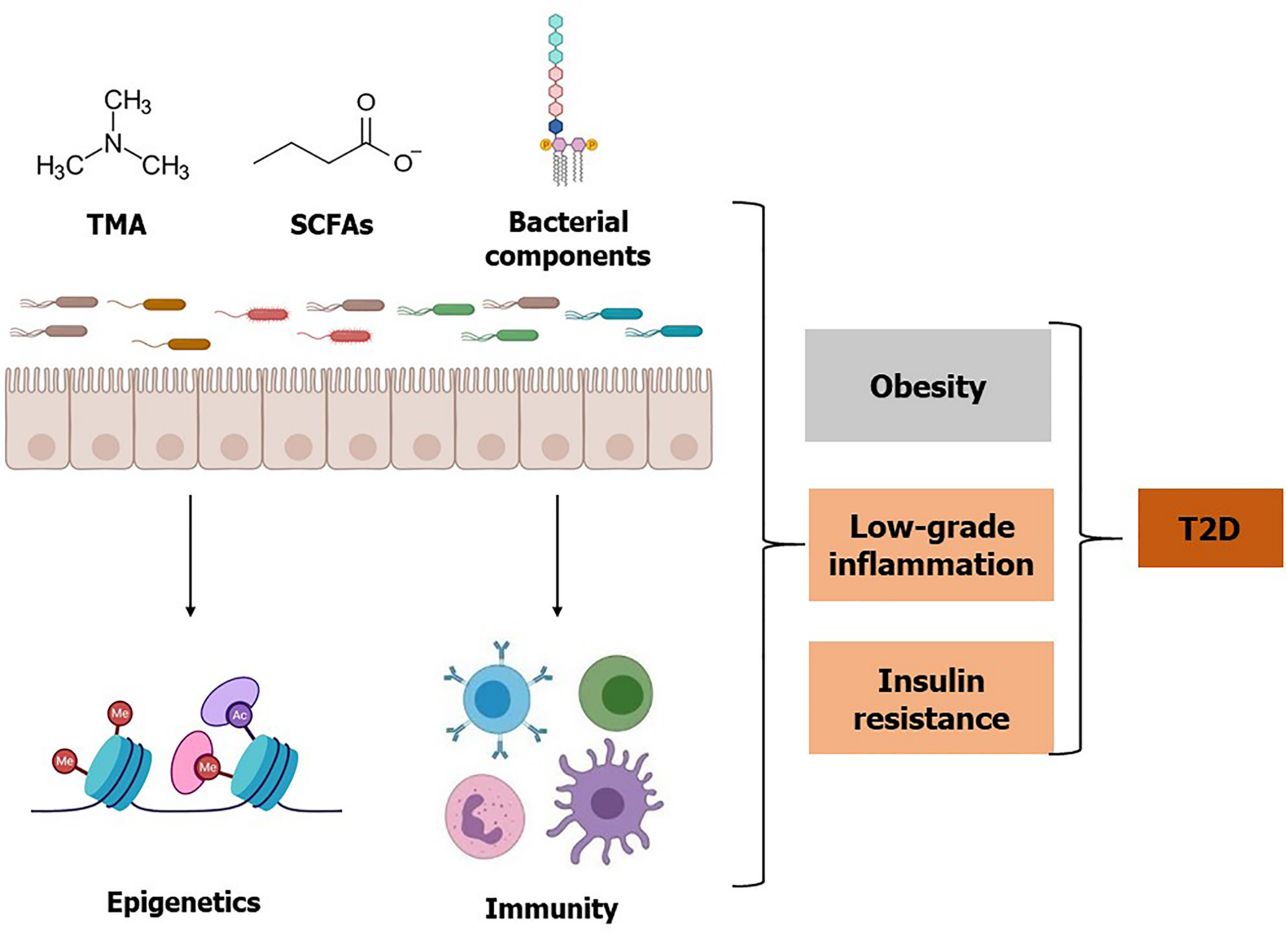
**Effects of gut microbiota, microbial metabolites, and bacterial components on T2D**. Gut microbiota and specific bacterial taxa are associated with a risk of obesity, low-grade inflammation, and insulin resistance. Microbial metabolite TMA is converted to TMAO by the host enzyme and elevated TMAO is associated with insulin resistance. Whereas some bacterial metabolites such as SCFAs may improve glucose homeostasis. Additionally, SCFAs influence epigenetic programming by inhibiting histone deacetylase enzyme activity, which may improve insulin resistance and T2D. Besides live bacteria, bacterial components such as LPS, flagellin, and peptidoglycan can elicit an inflammatory response and may contribute to the increased risk of T2D. Conversely, some bacterial components such as Amuc 1100 can improve T2D. Referred studies can be found on the main body of this review.

Obesity and dysbiosis may cause low-grade inflammation (**Figure 2**) which also contributes to insulin resistance and the development of T2D. Several studies have demonstrated associations between gut microbiota, or microbial components, and low-grade inflammation in T2D (34). An array of bacterial components such as lipopolysaccharides (LPS) (35), flagellin (36), and peptidoglycan (37) can elicit an inflammatory response. LPS binds to immune cell receptors such as Toll-like receptors and Nucleotide Oligomerization Domain (NOD)-like receptors and triggers the expression of proinflammatory mediators that fuel chronic inflammation, promoting metabolic dysregulation and development of T2D (38). The interaction of specific microbes in the gut with the immune system is complex. Some gut bacteria and microbial components promote low-grade inflammation, while others stimulate anti-inflammatory cytokines and chemokines. For example, induction of interleukin (IL)-10 and IL-22 by species of *Roseburia*, *Bacteroides*, *Akkermansia*, and *Lactobacillus* (33, 39–43) may contribute to restoring insulin sensitivity and improving glucose metabolism (43, 44). Similarly, *Bacteroides thetaiotaomicron*, *Roseburia intestinalis*, *Clostridium* clusters *IV*, and *XIVa* induce T_reg_ cells (45, 46), which are tolerogenic immune cells and are important for maintaining a balance between pro and anti-inflammatory immune responses (47). Additionally, butyrate produced by the gut microbiota enhances colonic T_reg_ differentiation through epigenetic modification of histone deacetylase inhibition (48, 49) and is discussed in detail below along with other short-chain fatty acids (SCFAs). Inhibition of pro-inflammatory cytokines and chemokines is another pathway that beneficial microbes use to prevent low-grade inflammation. Various species of *Lactobacillus*, *Bacteroides, Roseburia*, and *Akkermansia* can decrease pro-inflammatory cytokines such as IL-1β, IL-6, IL-8, IL-17, and tumor necrosis factor (TNF)-α (40, 50–52). Conversely, *Fusobacterium nucleatum* and *Ruminococcus gnavus* can increase inflammatory cytokine production. Therefore, depending on the composition, the gut microbiota may contribute to increased or decreased low-grade inflammation, impacting insulin sensitivity and T2D.

Gut microbiota profiling performed on large cohorts of T2D patients has found the abundance of several bacteria enriched or depleted in T2D subjects compared to controls (**Table 1**). In general, T2D patients commonly have a decreased abundance of SCFA producing bacteria (*Eubacterium rectale*, *Faecalibacterium prausnitzii*, *Roseburia intestinalis*, *Roseburia inulinivorans, Akkermansia*, and *Bifidobacterium*) and tryptophan metabolite producing bacteria (*Lactobacillus, Bacteroides, Bifidobacterium, Peptostreptococcus, Ruminococcus, Ruminiclostridium*, and *Clostridium*), and an increased abundance of opportunistic pathogens (*Bacteroides caccae* and *Clostridium hathewayi)*, branch chain amino acid synthesizing bacteria (*Bacteroides vulgatus* and *Prevotella copri*), and sulfate-metabolizing bacteria (*Desulfovibrio*, *Lactobacillus gasseri*, and *Lactobacillus reuteum*) compared to healthy controls (17–22, 26, 53). However, we note that not all the data derived from observational studies have been consistent. For example, one study comparing significant differences in gut microbiota diversity between T2D patients and healthy individuals was conducted on fecal samples from 18 men (24). In this study, decreased *Clostridia* and increased *Bacteroidetes* and *Proteobacteria* were observed, while overall diversity of the gut microbiota was positively correlated with plasma glucose levels in T2D patients. However, these results have not been identified in three large-scale metagenomics analyses performed in Europe and China (17, 18). A decrease in *Prevotella* was observed in 50 Japanese T2D patients compared to the healthy subjects (53), but in studies of 291 Nigerians and 171 Chinese, increased *Prevotella* abundance was associated with T2D (23, 25). The reason for the discrepancy between studies may be due to a number of confounding variables such as diet, genetics, medication use, and sequencing techniques. Utilizing alternative approaches and developing new technologies are of critical need to determine which of the associations between the microbiota and T2D are causal and which of the microbial differences are responsive to T2D.

## Microbial Metabolites

Beyond the direct effects of bacteria in the microbiota or their components, such as LPS, we now appreciate that the microbiota is a metabolically active “organ” that produces small biomolecules. In the following section, we highlight two important bacteria-derived metabolites TMAO and SFCAs, and briefly comment on other metabolites important for T2D. Gut microbiota is involved in the digestion of food ingredients and produces metabolites supporting physiological function in the human body (54). Microbial metabolites assist our interpretation of the underlying mechanisms by which gut bacterial taxa influence host health and disease (**Figure 2** and **Table 2**).

**Table 2:** Role of microbial metabolites on T2D.

Metabolites	Metabolite production pathway	Metabolite-producing bacteria (genus)	Mechanism on T2D risk	References
TMAO	Choline (diet) -> TMA (intestine) -> TMAO (liver)	• TMA: *Anaerococcus, Clostridium, Desulfovibrio, Edwardsiella, Proteus, Providencia*, and others	- Impair glucose tolerance by mediating the insulin signaling pathway in the liver - Increased HOMA-IR, exacerbated the impaired glucose tolerance, and upregulate expression of pro-inflammatory mediators in adipose tissue	Qi et al. (55) Gao et al. (56)
SCFA (Acetate, propionate, and butyrate)	Fiber (diet) -> Acetate, propionate, and butyrate (intestine)	• SCFA: *Anaerostipes, Blautia, Coprococcus, Eubacterium, Faecalibacterium, Marvinbryantia, Megasphaera, Roseburia, Ruminococcus*, and others	- Improve glucose metabolism and energy homeostasis - Increase intestinal glucose production and epithelial barrier function by promoting epithelial growth and *Bacteroides* species - Regulate the intestinal immune system by binding GPR41, GPR43, and GPR109A - Reduce plasma glucose level, appetite, insulin secretion, and slow gastric emptying by stimulating GLP-1 and GLP-2 secretion	Morrison et al. (57) Hirasawa et al. (58) Hamer et al. (59) Ross et al. (60)
Imidazole propionate	Histidine (diet) -> Imidazole propionate (intestine)	• Imidazole propionate: *Citrobacter, Dickeya, Eggerthella, Lactobacillus, Pectobacterium Staphylococcus*, and *Streptococcus*	-Suppress insulin signaling by mediating the activation of signaling pathways and insulin receptor substrates including rapamycin complex 1 (mTORC1)	Koh et al. (61)
Tryptophan metabolites (tryptamine, indole, indolelactic acid (ILA), indolepropionic acid (IPA), indoleacetic acid (IAA), and skatole	Tryptophan (diet) -> tryptamine, indole, ILA, IPA, IAA, and skatole (intestine)	• All tryptophan metabolites: *Clostridium* • Tryptamine: *Ruminococcus* • ILA: *Lactobacillus* and *Bifidobacterium* • IPA: *Peptostreptococcus* • ILA, IAA, and skatole: *Bacteroides*	- Reduce plasma glucose level, appetite, insulin secretion, and slow gastric emptying by stimulating GLP-1 secretion - Enhance the intestinal epithelial barrier by acting on the pregnane X receptor - Stimulate gastrointestinal motility by stimulating serotonin secretion - Activate the immune system by acting on the aryl hydrocarbon receptor - Exert anti-inflammatory and anti-oxidative effects in the systemic circulation	Roager et al. (62) Dodd et al. (63) Venkatesh et al. (64)
Bile acids (BA)	Cholesterol (liver) -> Primary BA (liver) -> Secondary BA (intestine)	• Secondary BA: *Bacteroides, Bifidobacterium*, *Clostridium, Eubacterium, Lactobacillus*, *Listeria, Peptostreptococcus*, and *Ruminococcus*	- Bind with host nuclear receptors such as FXR (Farnesoid X receptor), PXR (Pregnane X receptor), vitamin D Receptor, RAR-related orphan receptor gamma, and G-protein coupled membrane receptor (TGR-5) and modulate insulin sensitivity, and gluconeogenic genes expression	Jia et al. (65) Chiang et al. (66) Zhou et al. (67)
Branched-chain amino acids (BCAA)	Glucose, amino acid (diet) -> BCAA (intestine)	• BCAA: *Lactobacillus*, *Leuconostoc*, and *Weissella *	- Interfere with insulin signaling *via* phosphorylation of insulin receptor substrate-1 (IRS-1) on serine residue by stimulating rapamycin and its downstream effector, mTOR/S6 kinase	Chen et al. (69) Mutaguchi et al. (173)

### Trimethylamine N-Oxide

Trimethylamine (TMA) is produced by intestinal microbial metabolism of dietary choline and carnitine and is transported to the liver *via* the portal vein. In the liver, TMA is converted to TMAO by the activity of flavin-containing monooxygenase 3 (FMO3) (70, 71). *In vivo* studies have identified several candidate microbial taxa associated with TMA/TMAO production including *Anaerococcus hydrogenalis*, *Clostridium asparagiforme*, *C. hathewayi*, *C. sporogenes*, *Desulfovibrio desulfuricans*, *Edwardsiella tarda*, *Escherichia fergusonii*, *Proteus penneri*, and *Providencia rettgeri* (55). TMAO concentrations are elevated in T2D patients, suggesting that this pathway is associated with T2D (72, 73). However, it is not yet clear if the elevated TMAO has a direct causal effect on T2D development or if it is a consequence of T2D. Animal studies have shown that TMAO consumption impairs glucose tolerance by mediating the insulin signaling pathway in the liver and upregulates the expression of pro-inflammatory mediators in adipose tissue (56). Reduction of plasma TMAO by FMO3 knockdown also decreases plasma glucose and insulin levels, whereas FMO3 overexpression increases plasma glucose level and induces insulin resistance (74). A similar relationship between TMAO and T2D may exist in humans as circulating TMAO concentration was found to be significantly higher in T2D patients compared to control subjects observed in a meta-analysis (75). In contrast, a recent Mendelian randomization analysis suggests that elevated circulating TMAO is a consequence of T2D not causal (76). Additional studies on the relationship between TMAO and T2D are needed to clarify these results.

### Short-Chain Fatty Acids

SCFAs are microbial metabolites produced in the colon and are known to have a wide range of biochemical effects on the host (77). *Anaerostipes*, *Blautia*, *Coprococcus*, *Eubacterium*, *Faecalibacterium*, *Marvinbryantia*, *Megasphaera*, *Roseburia*, and *Ruminococcus* are among the primary gut microbes that produce SCFAs. The SCFAs acetate and butyrate improve glucose homeostasis by inducing intestinal production of glucagon-like peptide-1 (GLP-1) and peptide YY (PYY). These peptides in turn stimulate insulin secretion, suppress appetite, and slow gastric emptying (78–80). GLP-1 is released from colonic enteroendocrine L cells that are distributed along the length of the intestinal epithelium and are in direct contact with the gut microbiota (81). Additionally, SCFAs can regulate the intestinal immune system through G protein-coupled receptors (GPCRs) such as GPR41 and GPR43 (82). Reduced abundance of SCFA producing bacteria was observed in T2D patients suggesting that this pathway is altered in T2D (17, 83). A clinical trial demonstrated that selective enrichment of SCFA producing bacteria, achieved by dietary fiber supplementation, was associated with lower hemoglobin A1c (HbA1c) levels and improved glucose metabolism (84). Stool samples collected prior to and following the intervention were then mechanistically tested *via* adoptive transfer experiments using mice to establish causality (84). Overall, SCFAs are involved in glucose and lipid metabolism *via* activation of SCFA receptors (85). Therefore, SCFA could be an intermediate phenotype by which microbiota provides a beneficial effect of T2D prevention.

Besides TMAO and SCFAs there are several other microbial metabolites such as tryptophan catabolites: tryptamine, indole, indolelactic acid (ILA), indolepropionic acid (IPA), indoleacetic acid (IAA), indoleacrylic acid (IA), indolealdehyde (IAld), and 3-methylindole (skatole) [reviewed in (62)]; bile acids: deoxycholic acid (DCA) and lithocholic acid (LCA) [reviewed at (65, 86)], histidine catabolite: imidazole propionate (Imp) (87), branched-chain amino acids [reviewed at (88)], and hydrogen sulfide (84) have been investigated in the context of T2D (89–92). These microbial metabolites are involved in the regulation of host metabolism, immunity, gene expression, and intestinal integrity, creating an important link between the gut microbiota, and insulin resistance and T2D development. Individually, these results are intriguing but cumulatively the results are complex and heterogeneous. Alterations in the experimental system, environment, diet, or even circadian rhythms (93) all have been implicated as sources of variation contributing to the heterogeneity in the literature. Of particular interest to this review, the overall variation in the microbiota composition was better captured by a 17-day diet history (94). Therefore, information from longitudinal sampling and metabolomics is required to identify precise and dynamic interactions between diet, microbiota, and host.

## Interaction Between Host Genetics and Gut Microbiota on T2D

In addition to environmental factors including gut microbiota and microbial metabolites, host genetic architecture is associated with T2D (13). Many studies demonstrate that host genetics influences the community structure of gut microbiota in humans. This opens several interesting hypotheses regarding host-microbe symbiosis and perhaps the microbiota as a mediating variable contributing to the missing heritability in GWAS. More specifically, the contribution of genetic polymorphisms associated with T2D may be partly mediated through gut microbiota. One could wonder if intervention in the gut microbiota may improve T2D in susceptible individuals. Here we provide some recent evidence implicating interactions between host genetics and the microbiome that affect T2D.

Human studies of monozygotic and dizygotic twins have demonstrated that host genetics contribute to the composition of the microbiota (12), by tolerating or rejecting several microbial taxa. For example, the abundance of *Bifidobacterium*, an important commensal bacterium for T2D, is associated with host genotype at the lactase gene locus (LCT, rs4988235, and rs1446585) (95). Individuals with the GG genotype have reduced lactase activity and harbor higher levels of *Bifidobacterium* in their gut. Mechanistically these individuals provide more lactose to the bifidobacteria for utilizing as an energy source, which enriches bifidobacteria in their gut. Establishing if an increase in *Bifidobacterium* due to LCT genotype affects T2D remains to be determined.

In addition to the LCT locus and *Bifidobacterium*, genetic studies are beginning to identify some bacteria associated with specific genetic loci. For example, a recent GWAS found an association between *Ruminococcus* and rs150018970 near the gene *RAPGEF1* (96). RAPGEF1 is a signaling protein that transduces signals from GPCRs, which are involved in the regulation of gastrointestinal tract physiology, such as metabolism, immune cell differentiation, and tissue repair (97). Similarly, another study (98) found a quantitative trait locus for *Butyricicoccus* at the locus of SLC5A11 (rs72770483), which encodes a sodium-dependent myo-inositol/glucose co-transporter protein (99). These studies underscore the influence of host genetics on gut microbial colonization. However, further studies will be needed to determine to what extent host genetics affects the gut microbiota and T2D.

In addition to interactions between the host genotype and microbiota composition, we are now beginning to appreciate that microbial metabolites can influence host gene expression through epigenetic mechanisms (15). Thus, there seem to be bi-directional interactions with effects on both the host and specific bacteria of the microbiome. For example, in various tissues, including proximal colon, liver and white adipose tissue, microbial metabolites such as SCFAs influence epigenetic programming by inhibiting histone deacetylase (HDAC) enzyme activity, (100), which promotes de-condensation and relaxation of chromatin and increases chromatin accessibility to transcription factors (101). In particular, *Faecalibacterium prausnitzii* is one of the most abundant anaerobic bacteria in the healthy human gut that produces butyrate. Butyrate targeted inhibition of HDAC1 may have anti-inflammatory effects and ultimately improve insulin sensitivity by downregulating the IL-6/STAT3/IL-17 pathway (102). Butyrate may also influence differentiation of Th17 and T_reg_ cells through enhanced Forkhead Box P3 (*Foxp3*) expression (102). In human adipose tissue, the epigenetic regulation of the expression of genes involved in glucose and energy homeostasis, such as insulin-like growth factor 2 mRNA binding protein 2(*IGF2BP2*), is associated with gut bacterial populations (103). These data support the idea that the gut microbiota could act as an epigenetic regulator in T2D (104). A genome-wide DNA methylation analysis of isolated human pancreatic islet cells harvested from donors with and without T2D revealed 853 unique differential DNA methylation genes, including 17 genes previously identified in GWAS such as *TCF7L2*, *THADA*, *KCNQ1, FTO*, and *IRS1* associated with the risk of T2D (105). This reinforces the idea that genetic and epigenetic mechanisms may interact to affect pancreatic β-cell function, development of insulin resistance, and T2D. Understanding if the microbiota specifically aids the host epigenetic changes associated with T2D could be important in the development of novel therapies T2D or comorbidities such as obesity.

## Therapeutic Potential of Gut Microbiota for T2D

The associations between gut microbiota, microbial metabolites, and T2D, opened a new perspective for potential novel therapeutics for T2D. Several gut microbiota targeted therapeutics including fecal microbiota transplantation (FMT), medication, and dietary choices could be useful therapeutic strategies to manage T2D (**Figure 1**). Several clinical trials to evaluate the impact of these potential therapeutic agents on T2D are currently completed or in progress (**Table 3**).

**Table 3:** Ongoing or completed clinical trials on T2D with FMT, medication, prebiotics/functional foods, or probiotics.

Category	NCT Number	Title	Interventions	Country	Age	Phases	Enrollment
FMT	NCT02346669	Fecal Microbiota Transplantation for Diabetes Mellitus Type II in Obese Patients	FMT	Israel	18–65	Phase 2	30
	NCT01790711	Fecal Microbiota Transplantation on Type 2 Diabetes Mellitus	FMT	China	18–70	Phase 2| Phase 3	30
	NCT03127696	Randomized Placebo-controlled Study of FMT to Impact Body Weight and Glycemic Control in Obese Subjects With T2DM	FMT	China	18–70	NA	61
Medication	NCT03018444	The Effect of HMG-CoA Reductase Inhibition on Postprandial GLP-1 Secretion	Atorvastatin	Denmark	18–70	NA	15
	NCT02900417	Evaluation of the Effect of Sitagliptin on Gut Microbiota in Patients With Newly Diagnosed Type 2 Diabetes	Sitagliptin	China	40–70	NA	9
	NCT02061124	Effect of Bile Acid Sequestration on Postprandial GLP-1 Secretion, Glucose Homeostasis and Gut Microbiota	Sevelamer 1600 mg for 7 days	Denmark	35–80	NA	50
	NCT02960659	Therapeutic Targets in African-American Youth With Type 2 Diabetes	Metformin and Liraglutide	USA	12–25	Phase 1	92
	NCT04426422	Effect of Metformin on Gut Microbiota Changes and Glycemic Control of Newly Diagnosed Type 2 Diabetes	Metformin Hydrochloride	China	18–65	Phase 4	52
	NCT01758471	Efficacy of Acarbose on Intestinal Microbiome and Incretins of Type 2 Diabetes	Glipizide | Acarbose	China	40–60	Phase 4	160
	NCT04057261	Effect of Liraglutide on the Metabolic Profile in Patients With Type 2 Diabetes and Cardiovascular Disease	Liraglutide	Germany	18–	Phase 3	50
	NCT02583438	Evaluate the Effect of Saxagliptin on Gut Microbiota in Patients With Newly Diagnosed Type 2 Diabetes	Saxagliptin	China	20–65	Phase 4	100
	NCT04287387	Response of Gut Microbiota in Type 2 Diabetes to Hypoglycemic Agents	Glucophage | Acarbose | Sitagliptin | Dapagliflozin | Pioglitazone | Glimepiride Tablets	China	18–65	Phase 4	180
Prebiotics/ Functional foods	NCT03557541	Sardine-enriched Diet for Prevention Type 2 Diabetes	Sardine diet	Spain	65–	NA	182
	NCT03708887	The Effect of Omega-3 FA on Glucose and Lipid Homeostasis Disorders in Obese/Diabetic Patients	Omega-3 fatty acid		50–70	Phase 4	900
	NCT03194152	Peanut Consumption and Cardiovascular Disease Risk in a Chinese Population	Peanut	USA	20–65	NA	238
	NCT04403217	Effect of MEDiterranean Diet on the microBIOME of Individuals With Type 2 Diabetes	Individualized structured dietary plan	Portugal	40–80	NA	30
	NCT02294526	A Sardine Diet Intervention Study to Assess Benefits to the Metabolic Profile in Type 2 Diabetes Mellitus Patients	Sardine diet	Spain	40–85	NA	35
	NCT02717078	The LoBAG Diet and Type 2 Diabetes Mellitus	Diet Therapy	USA	18–	NA	50
	NCT03120299	The Effect of Omega-3 FA on Hypertriglyceridemia in Patients With T2DM(OCEAN)	Omega-3 fatty acid	China	20–75	Phase 4	350
	NCT02929901	The Effects of Coffee Main Constituents (Caffeine and Chlorogenic Acid) Supplementation on Inflammatory, Metabolic Factors, Hepatic Steatosis and Fibrosis in None- Alcoholic Fatty Liver Patients With Type 2 Diabetes	Caffeine and chlorogenic acid	Iran	30–65	Phase 2| Phase 3	200
	NCT03141710	Commercial Prebiotic Supplement Study	Prebiotics	Scotland	18–65	NA	12
	NCT03552991	Effects of Dietary Fiber on Glucose Control in Subjects With Type 2 Diabetes Mellitus	Agiocur Pregranules	South Korea	50–	Phase 4	14
	NCT02974699	Role of Gastrointestinal Microbes on Digestion of Resistant Starch and Tryptophan Availability to Humans	Potato Starch | Pregelatinized Starch	USA	18–65	Early Phase 1	20
Probiotics	NCT01765517	Study to Explore the Effects of Probiotics on Endotoxin Levels in Type 2 Diabetes Mellitus Patients	Probiotics	Saudi Arabia	20–75	NA	83
	NCT02728414	Probiotics Effect on Glucose and Lipid Metabolism and Gut Microbiota in Patients With Type 2 Diabetes	Probiotics	China	20–80	NA	100
	NCT04089280	Probiotics in Metformin Intolerant Patients With Type 2 Diabetes	Sanprobi Barrier-multispecies probiotics	Poland	18–75	NA	50
	NCT03037918	Effect of Yakult Ingestion on Diet-induced Insulin Resistance in Humans	Yakult light	England	18–30	NA	56
	NCT01250106	Probiotics as a Novel Approach to Modulate Gut Hormone Secretion and Risk Factors of Type 2 Diabetes and Complications	*Lactobacillus reuteri*	Germany	40–65	Phase 1| Phase 2	20
	NCT04495972	Intestinimonas for Prevention of Type 2 Diabetes Mellitus	*Intestinimonas*-capsules	Netherlands	18–65	Early Phase 1	26
	NCT01836796	Metabolic Effects of Lactobacillus Reuteri DSM 17938 in Type 2 Diabetes	*Lactobacillus reuteri*	Sweden	50–75	NA	46
	NCT04296825	Effect of Camel Milk With Probiotic on Type 2 Diabetes Mellitus	Camel milk containing *Bifidobacterium animalis A6* | Camel milk | *Bifidobacterium animalis A6* | Cow milk	China	35–68	Phase 1	45
	NCT02861261	A Study on the Efficacy and Gut Microbiota of Berberine and Probiotics in Patients With Newly Diagnosed Type 2 Diabetes	Berberine hydrochloride tablets and ProMetS probiotics powder	China	20–69	Phase 3	400
	NCT00699426	The Effect of Nexium and Probiotics on Insulin Secretion and Cardiovascular Risk Factors in Patients With Type 2 Diabetes	Nexium | Yoghurt	Denmark	40–70	Phase 3	41
	NCT03377946	Effect of Probiotics on Pre-diabetes and Diabetes in China	Probiotics	China	18–60	NA	220
	NCT01752803	RCT Examining Effects of Probiotics in T2DM Individuals	Probiotics	Malaysia	30–65	NA	100
	NCT01620125	Metabolic Control Before and After Supplementation With Lactobacillus Reuteri DSM 17938 in Type 2 Diabetes Patients	*Lactobacillus reuteri*	Sweden	50–80	Early Phase 1	12

T2D, type 2 diabetes; NAFLD, nonalcoholic fatty liver disease; FMT, fecal microbiota transplantation; HbA1C, hemoglobin A1C; IR, insulin resistance; HOMA, homeostasis model assessment; HDL, high-density lipoprotein; LDL, low-density lipoprotein; TNF-α, tumor necrosis factor-alpha; IL, interleukin; CRP, C-reactive protein; OGTT, oral glucose tolerance test. (Data from https://clinicaltrials.gov).

### Fecal Microbiota Transplantation

FMT has gained attention over the past few years as a research method demonstrating the contribution of gut microbiota to a disease state. Most clinical trials with FMT have been performed in patients with *Clostridium difficile* infections (106, 107) and these studies have been successful. As an extension of these studies, several additional diseases such as T2D have been suggested responsive to microbiota transplantation (108). In rodent models, insulin sensitivity significantly improved after transferring microbiota in MyD88 deficient NOD mice (109). Similar studies where human microbiota from healthy Chinese subjects are transplanted into diabetic db/db mice remarkably lowers fasting blood glucose concentrations (110). Likewise, transplantation of fecal samples of patients treated with metformin into germ-free mice improves glucose tolerance (111). A limited number of studies have begun to suggest that FMT from lean subjects into patients improves insulin sensitivity which could be in part due to increased butyrate-producing bacteria (108). One study examined the effects of lean donor versus self-FMT on metabolic syndrome patients and found that insulin sensitivity improves significantly at 6-weeks after FMT in male recipients with the metabolic syndrome (112). However, FMT treatment sometimes failed to improve targeted clinical phenotypes. For example, one study failed to show reduced TMAO levels in the recipient of FMT from a vegan donor (112), who have altered intestinal microbiota compared to omnivores (114) and low production of TMAO (115). In addition to inconsistent results, the long-term effects of FMT have not been adequately examined. Thus, further studies are needed to evaluate the long-term effectiveness and potential side-effect of FMT in humans.

### Anti-Diabetic Drugs

Metformin is a widely known common treatment for T2D but the exact mechanisms underlying the hypoglycemic effect are not yet fully understood. Metformin has been shown to have an inhibitory effect on T2D by activating AMP-activated protein kinase (AMPK) or inhibiting mitochondrial respiration and glycerophosphate dehydrogenase (116–118). Recently, evidence has been reported suggesting that the composition of the gut microbiota mediates the efficacy of metformin to lower blood glucose levels. The fact that intravenous injection of metformin, unlike oral administration, does not lower hyperglycemia, suggests that gut microbiota is an important part of metformin action (119). Indeed, metformin shifts the composition of gut microbiota in both mice and humans, making them more similar to the microbiota of a healthy host (111, 120, 121). Some of these gut microbiota changes have also been seen in healthy people who have not responded to glycemic control to metformin treatment, thus suggesting shifts in the gut microbiota induced by metformin itself, rather than simply reflecting lowered blood glucose level. Metformin influences the abundance of several microbial taxa, including increased abundance of *A. muciniphila*, *Bifidobacterium bifidum*, *Bilophila wadsworthia*, *Escherichia*, *Lactobacillus, Shigella* spp. as well as a reduced abundance of *Clostridium* spp. and *Intestinibacter* spp. (111, 122, 123). Regarding the effects of these changes on blood glucose, metagenomic analysis of microbial composition demonstrates changes in various functional pathways affecting the production of propionate and butyrate (124, 125). Metformin stimulates the activity of endocrine cells by regulating bile acid conversion, improving intestinal permeability, reducing endotoxin levels, and enhancing the release of GLP-1 and PYY peptides (126). Metformin also decreases the TMA level and the growth of bacteria that produce it in the gut, and thus the circulating TMAO level in mice (127). The fact that transferring the microbiota from metformin-treated mice improves metabolic traits in aged mice indicates that the shifts in the gut microbiota by metformin treatment are beneficial (111). The effect of the microbiota on the efficacy of metformin remains unclear as a recent study found that metformin’s ability to improve T2D in mice was not affected by the elimination of gut microbiota using gnotobiotic mice or antibiotics (128). Although previous studies did not directly demonstrate the role of gut microbiota in improving glycemic control by metformin, it is suggested that the anti-inflammatory activity of metformin could potentially play a role in eliciting some beneficial effects regardless of the gut microbiota.

Another anti-diabetic drug with a link to the microbiota is Acarbose, an α-glucosidase inhibitor. Acarbose suppresses the conversion of oligosaccharides to monosaccharides and disaccharides, delays the absorption of glucose in the intestine, and lowers blood glucose levels after a meal. Due to its effects on carbohydrate metabolism, Acarbose has been hypothesized to affect microbiota composition. In T2D patients Acarbose treatment alters the gut microbiota. The abundance of *Dialister, B. longum*, *Faecalibacterium*, and *Lactobacillus* increases, while the abundance of *Butyricoccus*, *Phascolarctobacterium*, and *Ruminococcus* is reduced. These changes in composition may improve gut health as evidenced by the decrease in circulating LPS levels (129–131). This alteration in the gut microbiota composition after Acarbose treatment suggests that the therapeutic effect of Acarbose may be partially mediated through microbiota. Whether these changes in microbial composition contribute to acarbose’s effect on lowering blood glucose has not been extensively studied. Similarly, liraglutide, a GLP-1 receptor agonist, stimulates satiety, slows gastric emptying, inhibits glucagon, and promotes insulin secretion. In animal studies, liraglutide increased the abundance of *A. muciniphila, Allobaculum*, *Anaerostipes*, *Blautia*, *Butyricimonas*, *Desulfovibrio*, *Lactobacillus*, *Turicibacter*, and SCFAs producing bacteria and decreased the abundance of Bacteroidales, Clostridiales, Proteobacteria (132, 133). These data suggest that the beneficial effect on hyperglycemia these drugs have, may in part be through the gut microbiota, although further clinical studies are needed.

### Probiotics

Probiotics are live microorganisms that have a beneficial effect on human health (134). Various beneficial effects of taking probiotics have been reported, including improving gut health, alleviating symptoms of lactose intolerance, inhibiting the growth of pathogenic bacteria, producing SCFAs, balancing pH, and stimulating the immune system (135). The use of probiotics to manage T2D is of interest, but a limited number of studies have evaluated the effects in clinical settings. Preliminary studies indicated that alteration of the gut microbial composition by probiotics supplementation might improve T2D by reducing pro-inflammatory cytokines, intestinal permeability, and oxidative stress [reviewed at (136)]. Several bacterial species are used in commercial probiotics supplement products, including *Bifidobacterium longum* subsp*. infantis, Lactobacillus, Streptococcus, Pediococcus*, and *Lactococcus species* (137). *L. gasseri*, *Lactobacillus helveticus*, *Lactobacillus casei*, and *Bifidobacterium bifidum* probiotic reduce fasting blood glucose levels with HbA1c (138–140). Mechanistically, these probiotics have been shown to have antioxidant and immunomodulatory effects by reducing oxidative stress (140), reducing inflammatory molecules, and inhibiting effector functions of CD4^+^ T-cells (142), which may influence on the reducing blood glucose levels and T2D risk. A randomized, double-blind, placebo-controlled trial of administration of *A. muciniphila* in overweight/obesity insulin-resistant volunteers improved insulin sensitivity and reduced insulinemia, plasma total cholesterol, body fat mass, hip circumference, and level of blood markers associated with liver dysfunction and inflammation (32). Recent meta-analysis studies showed that the probiotic supplementation improved the fasting blood glucose, HbA1c, and homeostatic model assessment for insulin resistance (HOMA-IR) in T2D patients and thus can be recommended as complementary advice alongside medicine and lifestyle modifications for T2D treatment (143, 144).

### Prebiotics

Prebiotics are the non-digestible food ingredients that beneficially affect the host by selectively stimulating the growth and (or) the activity of one or a limited number of bacterial species already resident in the colon (145). Inulin, a linear β-2,1 fructosyl-fructose polydisperse carbohydrate material with or without a α-D-glucose moiety (146), is one of the most studied prebiotics. Inulin-type fructooligosaccharide (ITF) improved glycemia by increasing the production and release of the active forms of GLP-1 from the cecum and proximal colon and reducing plasma ghrelin concentration in the rat (147). The direct effect of inulin supplementation on the T2D is not conclusive in human clinical trials. One study reported that dietary inulin reduced fasting blood glucose, body weights, glycated hemoglobin, plasma LPS, IL-6, TNF-α and IL-17A in T2D patients (148). A recent placebo-controlled crossover clinical trial (149) found enrichment of *Bifidobacterium* and *Bacteroides* with a significantly higher fecal SCFAs concentration due to ITF consumption compared to placebo (150). Additionally, the relative abundance of *Cyanobacteria* and *Bacteroides* is increased, and a reduction in the relative abundance of *Ruminiclostridium*, *Deferribacteres*, and *Tenericutes* is observed due to inulin supplementation, indicating that the dietary inulin alleviates T2D *via* suppressing inflammation and modulating gut microbiota (148). A recent systemic review (151) has summarized clinical trials conducted to evaluate the effect of dietary inulin on *Akkermansia muciniphila*, which are usually present at a higher abundance in healthy individuals compared to T2D patients and found an increased abundance in the treatment group compared to controls. However, others found no effect largely due to interindividual variation at the baseline T2D phenotypes (152).

It should be noted that a symbiotic mixture of prebiotics and probiotics (134), supplementation could provide a better beneficial effect compared to prebiotic or probiotic alone (153). For example, *Lactobacillus acidophilus* DSM20079 induces 14·5-times more butyrate in the presence of inulin or pectin than glucose (154). Berberine, a natural plant alkaloid extracted from *Berberis aristata* and *Coptis chinensis*, is reported to be an effective remedy for T2D (155). A recent randomized, double-blind, placebo-controlled trial conducted in China demonstrates that administration of berberine with probiotics improves HbA1C levels compared to the group treated with berberine alone (156). A meta-analysis of randomized controlled trials reported that diets supplemented with either prebiotics or symbiotics improved fasting blood glucose and HbA1C in patients with T2D (157). Therefore, symbiotic products that selectively stimulate and (or) activate metabolism of probiotics could be recommended to effectively lower the risk of T2D.

### Intermittent-Fasting

Intermittent fasting (IF) is defined as a periodic dietary restriction, which has been shown to increase lifespan, and to reduce the risk of developing various age-related pathologies including T2D (158). Animal studies of IF have reported an improvement in body composition, glucose and lipid metabolism, decreased inflammation, and autophagy (159) and gut microbiota might play a pivotal role in this process (160, 161). Though most of the human IF studies show a beneficial effect, the results are not completely conclusive. Two recent reviews summarize the recent literature on the effect of IF on T2D (162, 163). In this portion of the review, we will critically evaluate the microbial aspect of the IF on T2D. A recent study (164) using diabetic mice reported that a 28-day IF intervention re-structured the gut microbiota by increasing the abundance of *Aerococcus, Corynebacterium, Odoribacter*, and *Lactobacillus* and decreasing the abundance of *Streptococcus*, *Rummeliibacillus*, and *Candidatusarthromitu*, which reduced plasma glucose and insulin levels, and improved energy metabolism. The changes in bacterial abundances due to IF are correlated with plasma secondary BAs concentration, increased villi length and reduced gut leakage accompanied by decreased plasma LPS levels (164), indicating improved low-grade inflammation (165). More importantly, the effect of IF on the T2D was suppressed by antibiotics treatment (164), suggesting that the microbiota is a causative agent of improvement in T2D by IF. An alternative to IF is a fasting-mimicking diet (FMD), which contains very low calories and low protein (166). Intermittent administration of FMD led to the reconstruction of gut microbiota by increasing the genera of *Parabacteroides* and *Blautia* while reducing *Prevotellaceae, Alistipes*, and *Ruminococcaceae*, along with normalized blood glucose levels, improved insulin sensitivity and β cell function in hyperglycemic db/db mice. This study further underscores that the loss of pancreatic islets and β cells can be prevented by the FMD-mediated altered gut microbiota (167), indicating that FMD improved T2D through pancreatic β cells function. Overall, IF may modulate gut microbiota and improve T2D. However, these findings need to be validated in human cohorts using longitudinal studies to establish the long-term effectiveness of IF in health outcomes including T2D.

## Concluding Remarks and Future Perspectives

Substantial evidence suggests the gut microbiota, and the metabolites it produces, are critical to the etiology of T2D. A strategy including FMT, medication, prebiotics, probiotics, functional food, and intermittent fasting has been suggested as strategies to reduce T2D. However, most studies have focused on the characterization of gut microbiota rather than functional validation of specific microbial taxa affecting T2D risk. Identifying specific causally related microbial taxa or microbial metabolites responsible for the pathogenesis of T2D could provide interesting new opportunities for the diagnosis, treatment, and prevention of T2D.

Recently several novel approaches have been taken to directly modify the gut microbiota. For example, one study (168) reported a novel approach to remodeling the gut microbiota using cyclic d,l-α-peptides. Alternatively, the FXR agonist fexaramine, which was not absorbed by the intestine, binds the FXR receptor on intestinal cells and induces enteric fibroblast growth factor 15 that leads to alterations in bile acid composition, reduces diet-induced weight gain, body-wide inflammation, and hepatic glucose production (169). These studies suggest that the development of therapeutics targeting the microbiome instead of the host is a viable strategy for T2D.

As discussed above, probiotic supplementation and FMT studies have established a causal relationship between gut microbiota and T2D. However, studies (170, 171) using FMT have demonstrated that the relationship between gut microbiota and disease phenotype is more complex than usual thought. For example, FMT may not always be able to transfer the beneficial clinical phenotype, instead sometimes can be resulted in a detrimental opposite effect. A recent FMT study (31) in which the gut microbiota of C57BL/6J mice ablated using antibiotics was reconstituted with either C57BL/6J or WSB/EiJ fecal microbiota. C57BL/6J mice are more susceptible to obesity, diabetes, and atherosclerosis compared to WSB/EiJ mice (170). Paradoxically, mice reconstituted with WSB/EiJ microbiota had significantly higher fat mass compared to the mice reconstituted with C57BL/6J microbiota. Moreover, among the members of gut microbiota, only the bacterial community is being studied extensively. To date, enteric virus, fungal, or archaea communities are still underappreciated mostly because of the assay difficulties and lack of standard reference databases and thus their contribution to T2D remains largely unknown. Therefore, incorporating these members in analysis may potentially lead to the development of novel therapeutics for T2D.

Computational approaches such as machine learning facilitate the analysis of large “-omics” datasets through the development of algorithms and mathematical models designed to predict outcomes. It remains to be determined how these novel computational approaches can be harnessed to further our understanding of the microbiota’s role in T2D but initial studies are promising. Recently two studies used machine learning tools to explore the role of the microbiome in precision nutrition (172) and to predict cirrhosis based on gut-microbiota features (173). Thus, use of these novel computational approaches may further our understanding of the metabolic consequences of how alterations in dietary habits, microbiota, metabolomics, genetics, and epigenetics, interact to alter metabolism. A better understanding of the interactions between microbiota, lifestyle, and host factors such as genetics and epigenetics might lead to a novel therapeutic approach for T2D.

## Author Contributions

BB supervised all portions of the review process, interpreted the results, and mentored manuscript writing. MH and MK conducted the literature search, extracting the information, and drafting the manuscript. MH and MK also addressed co-authors’ comments and concerns. BB, MH, and MK critically revised the manuscript. BB had primary responsibility for the final content. All authors contributed to the article and approved the submitted version.

## Funding

This research was supported in part by NIH grant 5R01HL128572 (BB), NIFA grant 2019-07731 (BB) and USDA project 2032-51530-025-00D (BB). The USDA is an equal-opportunity employer.

## Conflict of Interest

The authors declare that the research was conducted in the absence of any commercial or financial relationships that could be construed as a potential conflict of interest.
